# Immunohistochemical Profile of Parathyroid Tumours: A Comprehensive Review

**DOI:** 10.3390/ijms23136981

**Published:** 2022-06-23

**Authors:** Romans Uljanovs, Stanislavs Sinkarevs, Boriss Strumfs, Liga Vidusa, Kristine Merkurjeva, Ilze Strumfa

**Affiliations:** 1 Department of Pathology, Riga Stradins University, LV-1007 Riga, Latvia; romans.uljanovs@rsu.lv (R.U.); stanislavs.sinkarevs@rsu.lv (S.S.); boriss.strumfs@rsu.lv (B.S.); liga.vidusa@rsu.lv (L.V.); kristine.merkurjeva@rsu.lv (K.M.); 2 Latvian Institute of Organic Synthesis, LV-1006 Riga, Latvia

**Keywords:** parathyroid carcinoma, parathyroid adenoma, multiglandular parathyroid disease, atypical parathyroid tumour, WHO classification, immunohistochemistry, parafibromin, Ki-67, p27, calcium-sensing receptor, tumour microenvironment

## Abstract

Immunohistochemistry remains an indispensable tool in diagnostic surgical pathology. In parathyroid tumours, it has four main applications: to detect (1) loss of parafibromin; (2) other manifestations of an aberrant immunophenotype hinting towards carcinoma; (3) histogenesis of a neck mass and (4) pathogenetic events, including features of tumour microenvironment and immune landscape. Parafibromin stain is mandatory to identify the new entity of parafibromin-deficient parathyroid neoplasm, defined in the WHO classification (2022). Loss of parafibromin indicates a greater probability of malignant course and should trigger the search for inherited or somatic *CDC73* mutations. Aberrant immunophenotype is characterised by a set of markers that are lost (parafibromin), down-regulated (e.g., APC protein, p27 protein, calcium-sensing receptor) or up-regulated (e.g., proliferation activity by Ki-67 exceeding 5%) in parathyroid carcinoma compared to benign parathyroid disease. Aberrant immunophenotype is not the final proof of malignancy but should prompt the search for the definitive criteria for carcinoma. Histogenetic studies can be necessary for differential diagnosis between thyroid vs. parathyroid origin of cervical or intrathyroidal mass; detection of parathyroid hormone (PTH), chromogranin A, TTF-1, calcitonin or CD56 can be helpful. Finally, immunohistochemistry is useful in pathogenetic studies due to its ability to highlight both the presence and the tissue location of certain proteins. The main markers and challenges (technological variations, heterogeneity) are discussed here in the light of the current WHO classification (2022) of parathyroid tumours.

## 1. Introduction

Primary hyperparathyroidism [1,2,3,4], the classic manifestation of parathyroid tumours, represents the third most common endocrine pathology with an estimated prevalence of 3/1000 [5,6,7]. There is close bidirectional association between primary hyperparathyroidism and neoplasms of the parathyroid glands. In most cases, primary hyperparathyroidism is caused by parathyroid tumours. In turn, almost all parathyroid neoplasms present with primary hyperparathyroidism although the existence of non-functional parathyroid tumours, mainly carcinomas, has been suggested in few case reports [8,9,10]. Thus, the clinical and laboratory manifestations of the primary hyperparathyroidism represent the mainstay for the diagnostics of parathyroid tumours [1,2,3,4].

The epidemiological characteristics of parathyroid neoplasms are also largely derived from the data on primary hyperparathyroidism because most cancer registries concentrate on malignant entities while parathyroid tumours are predominated by adenomas. Indeed, adenomas are found in 80–85% patients affected by primary hyperparathyroidism. In 10–15% cases of primary hyperparathyroidism, multiple glands are involved; this condition was formerly known as primary hyperplasia [11]. Currently, the given pathology has been redefined by World Health Organization (WHO) as multiglandular parathyroid disease [12]. Parathyroid carcinoma is associated with 0.1% to 5% [11,13] of all cases of primary hyperparathyroidism. In the Western world, it is thought to be responsible for less than 1% of all cases [13] although significantly higher proportion of 5.2% (16 cases) has been reported in an Italian study of 290 surgically treated patients [14]. As the diagnosis of parathyroid carcinoma occasionally is reached only by morphological evaluation of surgical specimens, more frequent occurrence of malignancy could be expected among operated patients compared to the general group of primary hyperparathyroidism. However, the variability between surgical cohorts is also well-documented, e.g., parathyroid carcinoma constituted 2.1% vs. 0.3% of surgically treated sporadic primary hyperparathyroidism in two European cohorts [15]. In China, carcinoma constitutes 3.9% of primary hyperparathyroidism, affecting 14/361 operated patients [16]. The population incidence of parathyroid carcinoma is 3.5–5.7 cases per 10 million [17], and it is rising, e.g., from 3.8 to 6.6 per 10 million person-years in Korea over time period from 2003 to 2016 [18]. Parathyroid carcinoma represents 0.005% of total cancer burden [17].

Significant progress has been achieved in the diagnostics and treatment of parathyroid mass lesions. First, the growing awareness of parathyroid pathology and increased availability of laboratory and radiological evaluation have shifted the diagnostic paradigm from clinically based suspicion [11] or even difficult diagnosis [19] in symptomatic patients to almost incidental findings [20,21] via routine biochemical laboratory assessment of serum calcium and parathyroid hormone (PTH) levels. Indeed, the incidence of primary hyperparathyroidism raised sharply after standard serum calcium tests were invented [22]. The next surge of incidence has been associated with screening and in-depth evaluation of osteoporosis patients via bone density measurements in combination with assessment of calcium and PTH levels to identify secondary osteoporosis [7,22].

Second, the parathyroid surgery is currently benefitting from its golden age [21]. The indications, technologies and steps of operative intervention have been well-defined, supported by intraoperative assessment of parathyroid hormone. Currently, parathyroid surgery is considered safe and curative in 97–98% of cases [21].

Wider application of surgical intervention has expanded pathologists’ experience in diagnostic evaluation of parathyroid tissues. A stable basis for parathyroid research was set as well. This led to the third major achievement in parathyroid pathology—the current (2022) WHO classification that is based on deeper understanding of the pathogenesis of parathyroid disease, bringing at least three revolutionary innovations [12] in regard to (1) multiglandular parathyroid disease in primary hyperparathyroidism; as well as (2) atypical parathyroid tumour and (3) the novel concept of parafibromin-deficient parathyroid neoplasms.

Genetic and epigenetic changes drive the development of parathyroid neoplasms [23,24,25] and influence the proteome. To assess the presence and cellular location of certain proteins, immunohistochemistry is an indispensable tool. Due to its widespread use in diagnostic surgical pathology and abundant quality control programs, pathologists and technicians have reasonable experience with it, ensuring reliability. The method can be subjected to quantification via digital pathology and to technological standardization, e.g., via total test approach. Thus, immunohistochemistry has become a reasonable adjunct in pathology. For instance, the clinical significance of immunohistochemical surrogate tests in breast carcinoma is similar to gene expression profiling-defined molecular classification [26,27,28]; immunohistochemistry is studied as a substitute for molecular subtyping of gliomas [29,30] and develops as next-generation immunohistochemistry for detection of genetic alterations via evaluation of certain proteins [31].

**Figure 1:**
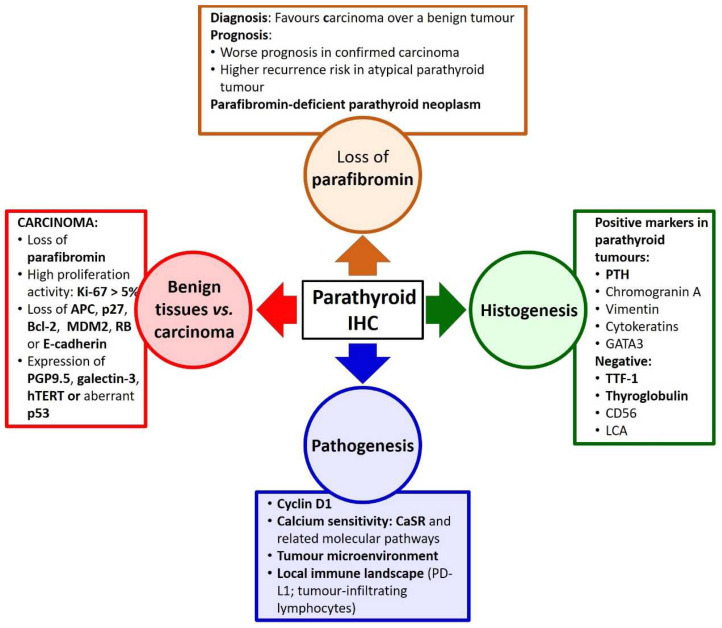
Applications of immunohistochemistry in parathyroid pathology.

In parathyroid pathology, immunohistochemical evaluation of parafibromin has an equally important role along with genetic detection of *CDC73* mutations. The other applications of immunohistochemistry in parathyroid disease (Figure 1) include pathogenetic studies (e.g., expression of cyclin D1 or PD-L1, or the cellular composition and molecular characteristics of the tumour microenvironment); ability of certain proteins and panels to distinguish between benign and malignant parathyroid tumours; and histogenetic differential diagnostic considerations (e.g., thyroid vs. parathyroid origin).

Considering the practical and scientific implications, the current review is devoted to immunohistochemical profile of parathyroid tumours in accordance with the new WHO (2022) classification of parathyroid neoplasms.

## 2. The Definitions: Morphological Diagnostic Criteria of Parathyroid Tumours by WHO Classification (2022)

To discuss the features of any clinicopathological entities, solid foundation is mandatory, namely, the definitions and diagnostic criteria, set by WHO and/or professional associations. This is particularly relevant to parathyroid tumours, as a new edition of WHO classification is released on 2022 [12], bringing some significant changes.

Historically, primary hyperparathyroidism was mostly attributed to three pathologies. The most frequent cause of primary hyperparathyroidism was parathyroid adenoma comprising 80–85% of cases; followed by primary parathyroid hyperplasia, found in 10–15% patients and the few cases of parathyroid carcinoma, responsible for less than 1% cases of primary hyperparathyroidism [32,33]. Upon typical presentation, these entities were easily recognised. Parathyroid adenoma was diagnosed if a single encapsulated or demarcated, non-invasive parathyroid neoplasm lacking intralesional adipose tissue was found in a patient experiencing surgery-related decrease of the parathyroid hormone level [34,35,36]. The diagnosis of adenoma was further supported by an adjacent peripheral rim of residual gland. Parathyroid hyperplasia presented as a multiglandular pathology showing mixture of parenchymal and fat cells with increased parenchyma-to-fat ratio [34,36,37]. Unequivocal invasive growth and/or presence of metastases justified the diagnosis of parathyroid carcinoma [38].

**Figure 2:**
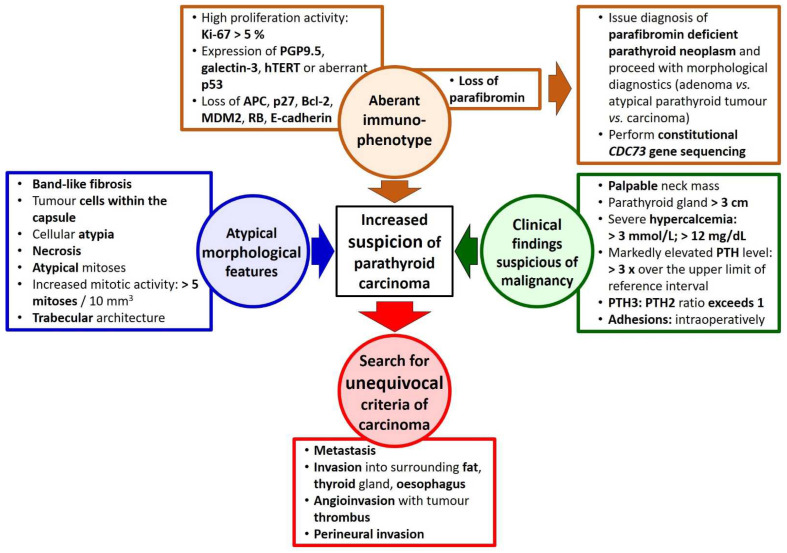
Suspicious features and unequivocal diagnostic criteria of parathyroid carcinoma.

However, the historical classification faced difficulties, which mainly focused on two areas: the clinically significant distinction between carcinoma and adenoma, and the differential diagnosis between adenoma and primary parathyroid hyperplasia. The problem “carcinoma vs. adenoma” would be triggered by a tumour that shows worrisome clinical, morphological or immunohistochemical features (Figure 2) yet lacks unequivocal invasion fulfilling the criteria of parathyroid carcinoma. To denote such tumours, the term “atypical parathyroid adenoma” was coined both in medical research [39,40,41] and informal hospital communications where it was used to emphasise the increased concern about the further course of disease, yet to avoid overtreatment and possible psychological insult associated with diagnosis of carcinoma.

The distinction between primary parathyroid hyperplasia and adenoma also had clinical relevance regarding the number of glands that should be surgically removed to cure the hyperparathyroidism. The pathological differential diagnosis could become difficult if no adjacent rim of normal or atrophic gland was present in adenoma or if nodular cell groups were evident in otherwise hyperplastic gland. In addition, the experiments in animal models [42] and immunohistochemical studies of human parathyroid tissue [38] suggested close pathogenetic relationship between so-called primary hyperplasia and adenoma. Further, the cases previously designated as primary parathyroid hyperplasia showed clonality contrasting to the expected polyclonal cellular proliferation in true hyperplasia [12].

Both these issues have been clarified in the current (2022) WHO classification of parathyroid tumours [12]. The entity of “atypical parathyroid tumour” should be used to classify former atypical adenomas that show certain suspicious features but still do not reach the established diagnostic criteria of carcinoma. Primary parathyroid hyperplasia involving multiple glands has been reclassified as multiglandular parathyroid disease [12], a term that is in line with surgeons’ needs to describe targets and approach [43]. Multiple multiglandular parathyroid adenomas is another recognised entity involving several parathyroid glands; the diagnosis is issued if each nodule corresponds to the features of adenomas [12].

Currently, the WHO classification of parathyroid tumours includes the entities of multiglandular parathyroid disease, adenoma, atypical parathyroid tumour, and carcinoma [12].

As previously, adenoma represents a benign tumour. It is well-circumscribed. In approximately 50% of cases, adjacent normal or atrophic glandular tissue is still present, contrasting with the tumour. Stromal fat is usually absent in adenoma; however, it can be abundant in lipoadenoma. Adenomas are composed of chief, oncocytic, transitional or water-clear cells. Follicles can be present; extensive follicular architecture must be distinguished from thyroid tissues via morphology or immunohistochemistry. Specific types of adenoma have been defined (Table 1).

Atypical parathyroid tumour is defined as a parathyroid neoplasm of uncertain malignant potential. It shows some cytological or histological features that increase suspicion of carcinoma, but the diagnostic criteria of carcinoma cannot be identified although sufficient number of tissue samples has been submitted for microscopy. The worrisome features that constitute the diagnostic criteria of atypical parathyroid tumour, include the following:

- **Trabecular** or **sheet-like** architecture;
- Band-like **fibrosis** in the absence of history of fine needle aspiration (FNA) that might induce scarring via needle track or at the site of FNA-induced necrosis. Secondary or tertiary hyperparathyroidism are also associated with fibrotic bands and should be considered clinically;
- Cytological **atypia**, enlarged nucleoli;
- **Mitotic activity** exceeding 5 mitoses/50 high power fields;
- **Atypical mitoses**;
- Coagulation **necrosis** in the absence of history of FNA;
- **Adherence** to the surrounding tissues but not frank invasion into these tissues;
- Tumour cells located **within the capsule**, but lacking full-thickness penetration through the capsule [12].

The presence of the listed traits should induce active search for the definitive criteria of parathyroid carcinoma. However, the characteristics of atypical parathyroid tumour themselves are not sufficient to justify the diagnosis of carcinoma.

Parathyroid carcinoma is a clear-cut malignancy, manifesting with either invasion or metastasis. To classify a parathyroid tumour as carcinoma, any of the following diagnostic criteria [12] must be present:

- **Angioinvasion in a blood vessel** located either outside the tumour or in the capsule; the tumour growth through vascular wall and/or carcinoma cells within thrombus must be visible. Considering the fenestrated endothelium, a mere presence of neoplastic cells in an intratumoural vessel does not qualifies for true invasion. Vascular invasion must also be distinguished from artificial displacement (“seeding”) of tumour cells into blood vessel lumen, that can happen during grossing. True vascular invasion is recognised by verified tumour penetration through vessel’s wall or by presence of the tumour cells in a thrombus, showing a biological reaction to invasion;
- **Invasion in lymphatics** provided that retraction phenomenon is excluded. Immunohistochemistry for endothelial markers is highly recommended for this;
- **Perineural or intraneural invasion**;
- **Invasion into surrounding soft tissues, thyroid, oesophagus, skeletal muscle**. Presence of neoplastic cells within the tumour capsule does not qualify for the diagnosis of carcinoma. The mere presence of parathyroid tissues within the thyroid also is not sufficient to justify the diagnosis of parathyroid carcinoma, because ectopic location of a parathyroid gland, adenoma or carcinoma is a well-known phenomenon [44,45]. Invasion must also be distinguished from parathyromatosis—a rare condition characterised by a presence of multiple microscopic islets of benign parathyroid tissue scattered throughout the soft tissues of neck and/or superior mediastinum [46,47,48,49,50];
- **Metastases** in lymph nodes or distant organs [12]. However, considering the indolent course of parathyroid carcinoma, metastatic spread is not always present. In a recently published large, SEER-based study, evaluating 609 cases of parathyroid carcinoma (1975–2016), lymph node metastases were found in 25.2% of all patients and 29.2% of cases where lymph node status was reported. Distant metastases were present in 2.2% of all patients and 3.8% of cases with a known stage [17].

## 3. Immunohistochemical Profile of Parathyroid Tumours

### 3.1. Parafibromin

Parafibromin, the tumour suppressor protein coded by *Cell Division Cycle 73* (*CDC73*) gene, represents the most extensively studied immunohistochemical target in parathyroid pathology. It is the driver of parathyroid carcinogenesis and thus the only protein that is advised to be detected immunohistochemically in parathyroid tumours (at least in all carcinomas and atypical parathyroid tumours) in accordance with the current (2022) WHO recommendations [12].

In 2002, germline mutation of *CDC73* gene, known also as *HRPT2* (hyperparathyroidism 2), was found in families affected by the autosomal dominant hyperparathyroidism-jaw tumour syndrome (penetrance 65–90%). This syndrome attracted attention due to the significantly increased lifetime risk of parathyroid carcinoma approaching 15% in mutation carriers [12,51,52].

As the name of syndrome “hyperparathyroidism-jaw tumour syndrome” indicates, carriers of germline mutation in *CDC73/HRPT2* gene have increased risk to develop hyperparathyroidism and ossifying fibromas of the maxillary and mandibular bones. The presence of parathyroid lesions and fibromas is reflected also in the name of parafibromin [51]. Regarding parathyroid pathology, single or multiple [35,39,53,54] parathyroid adenomas or parathyroid carcinomas are the most frequent features of this syndrome, seen in 90% of cases. Adenomas remain the most frequent cause of hyperparathyroidism even within the frames of hyperparathyroidism-jaw tumour syndrome [12]. However, the proportion of carcinoma is unusually high: in the hyperparathyroidism-jaw tumour syndrome patients, it is responsible for 15–37.5% of hyperparathyroidism cases [51] contrasting with the rare occurrence (0.1–5%) of carcinoma in the general cohort of primary hyperparathyroidism [11,13]. Hyperparathyroidism-jaw tumour syndrome can present as a seemingly sporadic parathyroid lesion. It has been estimated that 20–30% of apparently sporadic parathyroid carcinomas are associated with germline *CDC73/HRPT2* mutation [12,35,51]. Further, genetically confirmed hyperparathyroidism-jaw tumour syndrome can manifest with multiglandular parathyroid disease [54]. The frequency of benign fibro-osseous lesions (ossifying fibromas) of jaw bones is 10–30%; these lesions can be single or multiple, uni- or bilateral. In addition, renal cysts, hamartomas or tumours (Wilms tumour, papillary carcinoma, metanephric adenoma) are present in 10% of cases and heterogeneous spectrum of uterine pathology (reported as leiomyoma, adenomyosis, endometrial hyperplasia, adenofibroma or adenosarcoma)—in 40% of female patients. Occasionally, pancreatic adenocarcinomas, mixed germ cell tumours of the testis, Hurtle cell adenomas of the thyroid gland, and pituitary adenomas have been described in these kindreds [35,52,53,54].

However, the role of *CDC73/HRPT2* is not limited to a rare inherited syndrome. Somatic mutations of *CDC73* have been found in a significant fraction of sporadic parathyroid tumours: 60–90.9% of parathyroid carcinomas and up to 6% of parathyroid adenomas harbour *CDC73* mutation [39,41,51,55].

Parafibromin is a tumour suppressor protein that induces cell cycle arrest by repressing cyclin D1 [56]. It is involved in the regulation of p53 pathway [51]. *CDC73* mutations lead to loss of both function and immunohistochemical expression of parafibromin. Since the first discoveries, absence of parafibromin has been associated with diagnostic evidence [57,58,59] and worse prognosis of parathyroid carcinomas [59,60,61] and malignant behaviour of tumours histologically diagnosed as atypical adenomas [40]. However, controversies exist that can be attributed to technological differences and challenges [51], nuclear, nucleolar or cytoplasmic location of reactivity [62,63,64] or cases showing partial or weak expression [51,62,65,66].

In normal parathyroid glands, parafibromin is invariably present in the nuclei [67]. Loss of parafibromin has been reported in few cases (0–3.7%) of multiglandular parathyroid disease [38,67,68], 0–17.6% of adenomas [38,39,55,67] and 33.3–100% of parathyroid carcinomas [38,55,67,69] except carcinomas associated with tertiary hyperparathyroidism (0%) as reported by Tominaga et al., 2008 [70]. The main studies on parafibromin expression in parathyroid tumours are summarised in Table 2.

Technological difficulties represent the greatest problem of immunohistochemistry for parafibromin, followed and deepened by the differences in evaluation. The immunohistochemical results show overlap between adenoma and carcinoma. However, this is not attributable solely to technological shortcomings, but rather to the tumour biology as gene assessment also yields overlapping data. Mutations of *CDC73*/*HRPT2* have been reported in only 60–90.9% of parathyroid carcinoma and 1–6% of adenomas [39,41,51,55]. Clearly, the parafibromin profile overlap between adenoma and carcinoma is a part of parathyroid tumour biology.

In our experience, albeit the immunohistochemical stain is technically challenging, it has a rewardingly high diagnostic value. The procedure must be followed rigorously, and repeated stains can be necessary, but reliable final result with appropriate internal positive controls can be reached [38].

### 3.2. Proliferation Activity by Ki-67

Ki-67 is a nuclear protein that is expressed during the active phase of cell cycle while strongly down-regulated during the G0 phase. As the presence of immunohistochemically detectable Ki-67 identifies proliferating cells, Ki-67 is widely used in morphological protocols for tumour diagnostics, including grading, molecular classification, prognostic evaluation and prediction of treatment efficacy. The biological functions of Ki-67 include mitotic, interphase and regulatory processes. During mitosis, Ki-67 participates in the build-up of perichromosomal layer: a ribonucleoprotein sheath that coats the condensed chromosomes and prevents them from aggregation. In interphase, Ki-67 protein maintains the structure of heterochromatin. Ki-67 also regulates the cell cycle via p21 protein-related pathways [38,76,77].

The main studies on proliferation activity by Ki-67 in parathyroid tumours are summarised in Table 3.

Increased cellular proliferation by Ki-67 fraction has been shown in parathyroid tumours and hyperplasia in contrast to non-altered glands [81]. Further, statistically significantly higher proliferation activity was observed in parathyroid carcinomas than in adenomas [83]. The reported mean proliferation fraction in carcinoma ranges from 6.1% [82] to 8.4% [84] or even 13.9% [85]. In adenomas, the mean proliferation index by Ki-67 is reported as 1.9 [79]–4.3% [86] significantly exceeding the Ki-67 levels in residual parathyroid tissues [80]. Hence, currently it is generally believed that cut-off level at 5% can help to distinguish benign parathyroid tumours from the carcinoma although the sole proliferation fraction does not qualify for a WHO-accepted diagnostic criterion [12].

However, controversies still exist. Occasionally, the proliferation fraction in secondary parathyroid hyperplasia and multiglandular parathyroid disease has exceeded the values in adenoma and carcinoma [81]. Further, Kaczmarek et al. noted that normal and hyperplastic tissues were characterised by proliferation fractions of 3.5% and 1.8%, respectively [79]. In contrast, other studies have reported on increasing proliferative activity from normal glands to multiglandular parathyroid disease, adenoma and carcinoma [38]. 

Technological variables and shortcomings can affect any immunohistochemical visualisation procedure (Table 4). However, these issues are less probable regarding Ki-67 because it is a robust antigen, and most laboratories have extensive long-term experience in its detection. In parathyroid pathology, traps for Ki-67 assessment are set by the tumour biology. The most evident of them is the heterogeneity. Regarding Ki-67 expression, heterogeneity manifests as a prominent hotspot pattern: clustering of positive nuclei [38] strongly suggesting that proliferation and cell cycle regulation in parathyroid tumours also follows the principles of biological noise and positional effects [87]. Consequently, the mean and hotspot-measured highest proliferation fraction in tumours can differ significantly. For instance, the mean proliferation fractions in normal parathyroid glands, multiglandular parathyroid disease, adenomas and carcinomas are 0.4%; 1.0%; 1.6% and 5.8%, respectively; contrasting with hotspot-measured highest values in the same set of sections: 1.0%; 2.8%; 3.5% and 11.8% [38].

The differences between Ki-67 expression in various parathyroid pathologies retain statistical significance and range sequence between different pathologies irrespective of the mode of counting: mean vs. hotspot [38]. However, the numerical values and thus cut-off thresholds could differ. To avoid discrepancies, a unified evaluation protocol must be established setting the approach to the counting and the number of cells.

### 3.3. Cell Cycle Regulation

#### 3.3.1. p27 Protein

The p27 protein is best-known as a cyclin dependent kinase inhibitor and tumour suppressor that slows cell cycle progression, mediating G1 arrest. It also regulates G2/M progression. Other functions of p27 include control of cellular differentiation, motility and migration, as well as the activation of apoptosis. Malignant cells can lose p27 expression due to impaired synthesis or accelerated degradation, or inappropriate intracellular localisation of the relevant protein [88,89,90].

In parathyroid pathology, loss of p27 is considered an alert to possible malignancy. The published data almost invariably highlight low expression in parathyroid carcinoma despite slight controversies regarding benign parathyroid pathologies. Thus, decreasing levels of p27 expression were reported in normal parathyroid glands, hyperplastic tissues, parathyroid adenoma and carcinoma, namely, 89.6%, 69.6%, 56.8% and 13.9% by Erickson et al., 1999 [84]. Later, the expression levels were reported to be similar in adenoma and multiglandular parathyroid disease, but the down-regulation in parathyroid carcinoma was re-confirmed as statistically significant (*p* = 0.010). The biological differences also were marked. In normal parathyroid glands, almost all cells (97.9%) expressed p27 protein. The fraction of p27-expressing cells was 94.3% in multiglandular parathyroid disease associated with primary hyperparathyroidism and 92.8% in adenoma, contrasting with 59.0% upon malignant change [38]. Suppression of p27 in carcinoma has been verified by Arvai et al., 2012 [91], and the difference between adenomas, tumours of uncertain malignant potential and carcinomas (80% of adenomas vs. 43% of atypical adenomas vs. 18% of carcinomas by cut-off threshold at 30% of tumour cells) was found to be statistically significant [92].

Immunohistochemical downregulation of p27 protein represents one of the least controversial features of aberrant immunophenotype, that points towards diagnosis of carcinoma in a parathyroid tumour. Nevertheless, loss of p27 is also seen in parathyroid pathology within the frames of multiple endocrine neoplasia (MEN) syndromes [93].

#### 3.3.2. p21 Protein

The p21 protein controls cell cycle progression, apoptosis and transcription. It is the key mediator of cell cycle arrest in response to DNA damage [94] and a component of p53 pathway [92]. The expression of p21 has dual effects, including suppression or enhancement of apoptosis [94,95].

In early studies, setting the cut-off threshold at the level of 10%, nuclear expression of p21 was found in 58% of adenoma and 55% of carcinoma cases [92]. Tissue microarrays were used in the given study [92]. Later, significant heterogeneity of p21 expression was observed manifesting as the hotspot pattern [38]. The remarkable heterogeneity hinted on cautious interpretation of microarray-based results although the differences and trends in p21 expression were preserved independently of the counting mode: mean vs. hotspot [38].

The data provided by Stojadinovic et al., 2003, indicated similar p21 levels in adenoma and carcinoma [92]. More recently, comparison of mean and highest fraction of p21-positive cells disclosed statistically significant and biologically notable differences in p21 expression. The mean values were 3.1% in normal glands; 12.8% in adenoma; 15.7% in multiglandular parathyroid disease and only 7.6% in carcinoma (*p* < 0.001). The same pattern was followed by hotspot-measured highest values: 3.8% in normal glands, 23.7% in adenoma, 29.8% in multiglandular parathyroid disease and 15.6% in carcinoma (*p* < 0.001). Pathogenetically, these findings indirectly indicate either the duality of p21 [95] or a protective action that is up-regulated in early benign proliferations but lost upon malignant change. From the diagnostic point, the intermediate values in carcinoma do not encourage to use p21 for differential diagnosis between benign vs. malignant parathyroid disease. Finally, if changing p21 levels by targeting its translational regulation or post-translational modification will be considered as an additive therapy for specific cancers to suppress neoplastic phenotypes or to reduce drug resistance [95], in parathyroid pathology, multiglandular parathyroid disease, e.g., in relevant MEN syndrome patients could be the best-suited target.

#### 3.3.3. Cyclin D1

The cyclin D1 regulates transcription and acts as an important molecular switch in the proliferation control. As an allosteric activator, it forms a complex with cyclin dependent kinases 4 and 6 (CDK4 and CDK6) that phosphorylate and thus inactivate the tumour suppressor protein Rb, resulting in the cell cycle progress from the G1 to S phase [96,97]. The overexpression of cyclin D1 in parathyroid neoplasms can be caused by pericentric inversion of chromosome 11p that results in *CCND1* gene control by parathyroid hormone gene promoter. However, this inversion is seen in lower frequency than the overexpression of the relevant cyclin D1 protein, e.g., 5–8% vs. 40% in adenomas [98]. Consequently, other mechanisms are involved, such as gene amplification, transcriptional activation, e.g., via Wnt or MAPK pathways [96] or deranged degradation [99,100].

In transgenic mice, overexpression of the cyclin D1-coding gene resulted in hyperparathyroidism. This pathogenetic association was consistent with the primary role of cyclin D1 in parathyroid hyperfunction. Morphologically, the animals developed hyperplasia as well as asymmetrical encapsulated nodular growths that showed tubular architecture and compressed the adjacent gland, thus closely resembling adenomas. By immunohistochemistry, no cyclin D1 expression was found in parathyroid tissues of wild-type animals while irregular positive staining was evident in hyperplastic glands of transgenic mice [42].

Paralleling animal experiments, high levels of cyclin D1 protein have been reported in human multiglandular parathyroid disease in the setting of primary hyperparathyroidism. The highest fraction of cyclin D1-expressiong cells in multiglandular parathyroid disease exceeds the levels seen in adenoma hypothetically suggesting that cyclin D1 represents as an early molecular driver in parathyroid cell proliferation [38]. This is also in accordance with the fact that cyclin D1 stain lacks the ability to distinguish between benign vs. malignant parathyroid tumours [12]. Instead, it may show expression differences between the early stages: multiglandular parathyroid disease, formerly designated hyperplasia, and adenoma. These differences are statistically significant and biologically notable in contrast with minor margin regarding Ki-67 [38].

Expression of cyclin D1 shows remarkable intertumoural heterogeneity, both in adenomas and carcinomas [41,55] as well as significant intralesional heterogeneity with presence of cold and hot spots that closely resembles the patterns of Ki-67 and p21 expression. The set-up of scoring protocols (mean vs. highest fraction of cyclin D1-positive cells) can influence the degree of statistical significance and thus lead to different conclusions [38].

Several scientific teams have evaluated immunohistochemical expression of cyclin D1 in parathyroid carcinoma (Table 5). Truran et al. defined negative staining for cyclin D1 as the carcinoma-associated pattern. This feature was observed in a minor fraction of parathyroid carcinoma cases (2/24; 8.3%), and authors did not recommend to include it in the diagnostic panel of parathyroid carcinoma [57]. Several other research groups evaluated the contrary pattern: nuclear overexpression of cyclin D1 either by mean fraction of positive cells or by different cut-offs. However, they also disregarded overexpression of cyclin D1 as a marker for differential between parathyroid carcinoma versus adenoma [92,101].

Although higher levels of wild-type parafibromin have been shown to block expression of cyclin D1 [102], by immunohistochemistry, no correlation has been reported by expression of cyclin D1 and loss of parafibromin in parathyroid pathology [38,41,55,103].

**Table 5:** Expression of cyclin D1 in parathyroid tumours and tissues.

Pattern	Absolute Numbers of Pattern-Showing/Investigated Cases; Proportion of Positive Cases (%) or Fraction of Positive Cells (%)					
	Parathyroid Carcinoma	Atypical Parathyroid Tumour	Adenoma	Multiglandular Parathyroid Disease	Normal Gland	Reference
Mean value of strong nuclear expression	31.5%		12.0%	24.8% PPH ^1^	10.1%	Uljanovs et al., 2021 [38]
Highest (hotspot) value of strong nuclear expression	41.8%		22.8%	42.5% PPH ^1^	11.9%	Uljanovs et al., 2021 [38]
Nuclear expression exceeding 5%	7/10; 70.0%	10/14 AA ^2^; 71.4%	5/21; 23.8%			Sungu et al., 2018 [79]
Lack of expression considered as the carcinoma-associated pattern	2/24; 8.3%					Truran et al., 2014 [57]
Nuclear expression exceeding 5%	2/11; 18.2%	1/8 AA ^2^; 12.5%	4/44; 9.1%			Stojadinovic et al., 2003 [92]
Strong nuclear expression exceeding 20%	2/2; 100.0%		11/17; 64.7%		0/10; 0.0%	Thomopoulou et al., 2003 [81]
Mean value of strong nuclear expression	27.4% in joint group of carcinoma and adenoma		27.4% in joint group of carcinoma and adenoma	14.5% in PPH ^1^ 3.7% in SPH ^3^	<1%	Thomopoulou et al., 2003 [81]
Nuclear expression			41/46; 89.1%		9/10; 90.0%	Cristobal et al., 2000 [104]
Mean value of nuclear expression			25.8%		27.1%	Cristobal et al., 2000 [104]
More than 10% of cells in adenoma stained more intensively than non-tumour cells			9/24 (7, nuclear; 2, cytoplasmic); 37.5%			Ikeda et al., 2002 [105]

In the original sources, different terms have been used in accordance with the actual classifications and terminology at the time of publication: ^1^ PPH, primary parathyroid hyperplasia; ^2^ AA, atypical adenoma; ^3^ SPH, secondary parathyroid hyperplasia.

##### 3.3.4. p53 Protein

The “genome guard”, p53 protein is normally found within cells in small quantities due to a short half-life. The low physiological concentrations are almost undetectable by immunohistochemistry although some commercial antibodies stain wild-type p53 protein. *TP53* mutations can result in the synthesis of aberrant p53 proteins that have longer half-lives and therefore accumulate in cells reaching higher intracellular levels that become immunohistochemically detectable. On the other hand, silencing *TP53* mutations lead to absence of protein and therefore negative stain. Thus, *TP53* mutation analyses and immunohistochemistry for p53 protein provide two different levels of molecular assessment lacking correlation but providing complementary information [106].

Regarding p53 in parathyroid tumours, facilitated degradation of the relevant mRNA can be implicated. Parafibromin can bind to mRNA of p53 and destabilise it [64]. Enhanced association with mutant parafibromin [64] might result in faster degradation of p53 mRNA. The final outcome would be absence of immunohistochemically detectable p53 expression and enhanced cellular proliferation in parathyroid carcinoma while benign lesions retained wild-type protein. The general landscape of p53 expression in parathyroid diseases thus would lack diagnostic differences, remaining invariably negative. Indeed, constant negative p53 expression in normal parathyroid as well as benign and malignant tumours has been reported previously [38,92]. However, other research teams have noted reactivity in even 15% of adenomas [80] and overexpression of p53 in carcinoma [91]. Although reliable over-expression of p53 protein is still considered an alarming sign of possible malignancy [12], this feature seems to be rare in parathyroid carcinogenesis.

### 3.4. APC Protein

*Adenomatous polyposis coli* (APC) gene is a tumour suppressor that inhibits the Wnt molecular pathway. It is known for its role in colorectal carcinogenesis and association with familial adenomatous polyposis (FAP) [107,108]. Its protein product can be detected by immunohistochemistry and has been recommended by WHO (2022) as an adjunct in the diagnostics of parathyroid carcinoma [12]. Parathyroid adenomas usually retain APC while carcinomas tend to become negative, therefore loss of APC has been listed among the biomarkers that indicate an increased risk of malignant behaviour of a parathyroid tumour [12]. Hosny Mohammed et al. observed loss of APC in 20/21 (95.2%) parathyroid carcinomas, contrasting with 38/73 (52.1%) adenomas [69]. However, Kumari et al. reported on loss of APC (<10% of cytoplasmic staining) in 9% of carcinomas, 23.5% of atypical adenomas and 22% of adenomas [72]. Loss of APC acts as a screening marker for malignant potential, but the diagnosis of carcinoma still must be proved by WHO criteria, that are based on manifestations of invasive growth and metastatic spread.

Nevertheless, the information on APC in parathyroid tumours is quite scant. In a recent systematic review on biomarkers of parathyroid cancer [58], only five articles on APC levels (detected via immunohistochemistry or polymerase chain reaction) were included. In two publications, the APC expression in parathyroid carcinoma was found to be statistically significantly decreased. The third team noted a statistically insignificant up-regulation in carcinoma, and statistical evaluation was not performed in the remaining two articles [58].

### 3.5. Intermediary Filaments

#### 3.5.1. Cytokeratin 19

Cytokeratin 19 is a widely expressed intermediary filament. It is invariably present in parathyroid adenomas, carcinomas [109] and normal parathyroid glands [110]. Recently, a statistically significant up-regulation of cytokeratin 19 was found in proliferating parathyroid lesions encompassing adenoma, multiglandular parathyroid disease and carcinoma. The expression was markedly heterogeneous [38].

From the point of view of surgical pathologist, it is important to remember that thyroid tumours and cancer metastases in cervical lymph nodes also are likely to express cytokeratin 19 [111,112,113,114,115]. Hence, the diagnostic value of cytokeratin 19 in parathyroid pathology is low but this antigen could rather evoke scientific interest because of its up-regulation in carcinoma. As the diagnostic criteria of parathyroid carcinoma reflect capacity for invasion and metastatic spread, the altered expression level of intermediate filaments might have pathogenetic importance.

#### 3.5.2. Vimentin

Vimentin is a major mesenchymal intermediate filament, controlling cellular motility, signalling and directional migration [116].

The glandular histology mostly precluded the researchers from in-depth assessment of vimentin in parathyroid tissues, except stroma [110]. In addition, the rarity of parathyroid carcinoma hampered the studies of epithelial-mesenchymal transition in parathyroid malignancies.

In early reports, vimentin expression in normal parathyroid glands was found to be restricted to stroma [110]. The limited data on adenomas confirmed stromal reactivity but remained controversial in regard to the presence of vimentin in parenchyma [35,110]. Expression of vimentin has been reported in parathyroid carcinoma-derived cell line exhibiting both epithelial and mesenchymal traits [117].

Recently, our team highlighted some new features: parenchymal expression, different patterns and up-regulation of vimentin in proliferating parathyroid lesions along with changes in its expression pattern. In normal glands, only perinuclear, highly heterogeneous vimentin expression was observed. The fraction of vimentin-positive parenchymal cells increased from 9.3% in normal tissues to 11.7% in multiglandular parathyroid disease, 19.3% in adenomas and 36.8% in carcinoma. Paralleling the scores of vimentin-positive cells, cytoplasmic reactivity appeared. In carcinomas, the cytoplasmic expression pattern was invariable. Multiglandular parathyroid disease and adenomas showed combination of both patterns, with predominantly perinuclear pattern in multiglandular parathyroid disease and tendency to more frequent cytoplasmic staining in adenomas. Heterogeneity was remarkable in all groups, but only both benign proliferating parathyroid pathologies showed nodularity of vimentin expression [38].

### 3.6. CD44

CD44 represents a family of integral cell surface glycoproteins. It is a single-span transmembrane adhesion molecule lacking kinase activity [118,119]. The main ligand of CD44 is hyaluronic acid that is abundantly present in extracellular matrix. The interaction between ligand-binding domain of CD44 and hyaluronic acid changes the conformation of the molecule resulting in the recruitment of adaptor proteins (ERM, Src, and others) to its intracellular domain. This, in turn, triggers downstream biological effects as proliferation, motility and migration, adhesion and invasion. CD44 is expressed during embryonic development, on mesenchymal cells and in carcinogenesis. In tumours, it frequently indicates poor prognosis and is recognised as one of the cancer stem cell markers [29,118,119,120,121,122].

Only few scientific teams have studied CD44 expression in parathyroid tumours. Focal, irregular expression of CD44 in normal parathyroid glands and adenomas was described by Zeromski et al., 1998 [123]. A decade later, contrasting data appeared as up-regulation of CD44 was found in primary and secondary hyperparathyroidism. CD44 was present in 13/27 (48.1%) of the abnormal glands, showing statistically significant difference (*p* = 0.03) from the immunophenotypic absence of CD44 in normal glands [124]. Still later, almost complete absence of CD44 was re-confirmed in 179 parathyroid cases, including normal glands, multiglandular parathyroid disease, adenomas and carcinomas [38]. Thus, CD44 has no significant role of the pathogenesis, differential diagnosis or prognosis of parathyroid pathology.

The lack of CD44 in parathyroid tissues and tumours might seem unusual considering that chromogranin A expression in parathyroid neoplasms indicates neuroendocrine differentiation [12], and several other neuroendocrine tumours express CD44, although it is not a specific neuroendocrine marker. Presence of CD44 has been reported, e.g., in pancreatic neuroendocrine neoplasms, pulmonary carcinoids and high-grade neuroendocrine carcinomas (small cell carcinoma and large cell neuroendocrine carcinoma), and medullary thyroid carcinoma [125,126,127,128,129]. The key to solve the putative discrepancy might be in embryology. Neural crest-derived neoplasms tend to be CD44-negative while CD44 expression is more consistent for endoderm-derived neuroendocrine tumours [130]. Development of parathyroid glands in humans parallels the embryogenesis in mice [131] involving endoderm of the third and fourth pharyngeal pouches that interacts with and receives molecular signals from the surrounding neural-crest-derived mesenchyme [132,133,134,135]. Neural crest mesenchyme also contributes directly to the development of cervical structures, including parathyroid glands as evidenced by unusual co-expression of Snail, Twist and E-cadherin in normal and benign parathyroid glands [36,136].

### 3.7. Neuroendocrine and Hormone Markers: Chromogranin A, Synaptophysin, CD56, PTH and TTF-1

Neuroendocrine and hormone markers are helpful to detect the histogenesis of a tumour or mass lesion. Parathyroid tumours occasionally have to be distinguished from thyroid neoplasms because of close anatomic relation [137] between both glands, including occasional intrathyroidal location of normal parathyroid gland or parathyroid carcinoma. The histogenetic diagnosis is difficult also in fine needle aspiration cytology [138].

The neuroendocrine differentiation in parathyroid tissues is limited, generally manifesting as isolated positive reaction for chromogranin A, that is observed in most (98%) cases [12,139,140]. The expression of synaptophysin is less frequent albeit variable: 11–100%, according to Li et al., 2014 and Yu et al., 2019 [109,140]. Insulinoma-associated protein 1 (INSM1) is absent [140] both from normal and pathological parathyroid tissues including multiglandular parathyroid disease in primary hyperparathyroidism, secondary hyperplasia, tertiary hyperparathyroidism, adenomas, atypical adenomas and carcinomas [140]. Neural cell adhesion molecule CD56 is another negative marker despite frequent expression in neuroendocrine tumours in other locations [38,141,142,143,144].

CD56 is a membrane glycoprotein, representing a member of the immunoglobulin superfamily. It is expressed on neural cells, NK and certain other types of lymphocytes, muscle fibres as well as in different neoplasms [145]. Considering parathyroid pathology, early reports indicated absence of CD56 in normal and neoplastic glands [123]. Few later studies have been devoted to CD56 in parathyroid pathology. Although occasional expression by luminal membrane was noted [146], a recent study [38] confirmed the absence of CD56 from parathyroid tissues and tumours (except perivascular nerve fibres). Thus, in controversial cases, CD56 expression in a cervical neoplasm would favour non-parathyroid origin of the tumour. Follicular thyroid adenoma and carcinoma [147], primary and metastatic neuroendocrine tumours [148,149,150], NK cell lymphomas [150], malignant plasma cell dyscrasias, especially multiple myeloma [145]; and alveolar rhabdomyosarcoma [151] represent just few examples of CD56-positive differential diagnoses. Notably, pulmonary small cell carcinoma also express CD56 [149].

A positive result for chromogranin A should be combined with the data on PTH expression in the removed tissues/nodule [137] because other chromogranin A positive tumours enter the differential diagnosis. Medullary thyroid carcinoma is positive for chromogranin A [152], and this differential diagnosis can be especially difficult due to manifold histological structure of medullary thyroid carcinoma. Medullary thyroid carcinoma expresses calcitonin (80%) and carcinoembryonic antigen CEA in association with negativity for thyroglobulin. TTF-1 stain can be positive in up to 80%, and PAX-8 in 75% of cases. Medullary thyroid carcinoma also frequently (90%) features calcitonin-containing stromal deposits of amyloid, therefore positive reaction via Congo red stain and apple-green birefringence under polarised light are of diagnostic significance. The amyloid deposits are metachromatic upon visualisation with crystal violet [152,153,154].

Paragangliomas co-express vimentin, chromogranin A and synaptophysin but lack cytokeratins and calcitonin [154,155,156]. Expression of second-generation neuroendocrine markers, e.g., ISL1 and INSM1, has been reported in abdominal (sympathetic) paragangliomas [157]. In our experience, the expression of vimentin in paraganglioma is more marked, extensive and homogeneous than in benign parathyroid disease. Paraganglioma features Zellballen architecture with S100-positive sustentacular cells—a feature that is not seen in parathyroid neoplasms. However, the sustentacular cells can be lost in metastasis [154,155,156,157].

Pulmonary small cell carcinoma can metastasize to cervical lymph nodes, and the differential diagnosis can be emphasized by hypercalcemia due to lung cancer [158,159], either via bone metastases or paraneoplastic syndrome [160]. This high-grade tumour features a variable expression of neuroendocrine differentiation markers, including CD56, chromogranin A, synaptophysin and insulinoma-associated protein 1 INSM1 [161,162]. In our experience, chromogranin A and CD56 are the most informative markers in pulmonary small cell carcinoma. Chromogranin A frequently shows perinuclear expression in the form of tiny but bright perinuclear dots. Expression of CD56 is remarkable for its stability as it is retained even in crushed specimens. CD56 in small cell carcinoma tends to be more intense than chromogranin A: the contrary to parathyroid gland. The proliferation fraction by Ki-67 is very high, usually 70–100% [162]. Nuclei show the diagnostic salt-and-pepper structure of chromatin, and morphology reflects a high-grade malignancy with extensive necrosis, Azzopardi phenomenon and high mitotic activity.

Regarding PTH, it is detectable immunohistochemically and almost always present in parathyroid tumours and tissues [140,163] while being absent from thyroid gland [163]. Thyroid transcription factor TTF-1 and thyroglobulin are negative in parathyroid cells [12], therefore these are valuable markers in the differential diagnostics between parathyroid and thyroid origin of a neoplasm/mass [12,109]. Of note, calcitonin can be positive in parathyroid tumours [93] compromising the differential diagnosis with medullary carcinoma.

### 3.8. Immunohistochemical Profile of Parathyroid Disease in MEN Syndromes: Menin

Multiglandular parathyroid disease is a typical component of certain multiple endocrine neoplasia (MEN) syndromes, namely, MEN 1, MEN 2A and MEN 4. Loss of menin is characteristic for MEN I, and decreased expression of p27—for MEN 4. However, menin is a technologically “difficult” antigen similarly to parafibromin, and loss of p27 protein is also seen in sporadic carcinomas [93].

### 3.9. Calcium-Sensing Receptor (CaSR) and the Associated Molecular Pathways

Most of parathyroid tumours present with hypercalcemia that is higher and therefore more frequently symptomatic in patients affected by parathyroid carcinoma, compared to benign disease. Non-functioning parathyroid carcinoma hypothetically exists but is exceptionally rare [9,10]. Although tumour weight is strongly associated with calcium and PTH concentration in blood [164], abnormal feedback and/or disturbed sensitivity to blood calcium levels could be expected in the neoplastic cells, and the dysfunction might be more marked in carcinoma. Indeed, diminished calcium-sensing receptor expression has been reported in parathyroid carcinoma but is rare in benign tumours [165]. Thus, 31% of carcinomas showed downregulation of CaSR, contrasting with adenomas and hyperplasia. In this study [166], only a single adenoma featured a “carcinoma-like” irregular or absent CaSR staining pattern (1/104 in a mixed group of adenomas, primary multiglandular disease, secondary hyperplasia and tertiary hyperparathyroidism) [166]. More recently, global loss of CaSR has been reported in 5/10 carcinomas while all adenomas (21) showed retained expression (*p* = 0.001), and only a single atypical adenoma (1/14) yielded global loss of expression [66]. In contrast, Storvall et al. observed retained immunohistochemical CaSR expression in all the evaluated parathyroid tumours, including 32 carcinomas, 44 atypical adenomas and 77 adenomas; just a single carcinoma and one atypical adenoma presented weaker expression [165]. CaSR shows negative correlation with Ki-67 both in secondary hyperparathyroidism and adenoma [166,167,168].

Scaffold protein filamin A binds to calcium-sensing receptor and activates the mitogen-activated protein kinase MAPK pathway. Cytoplasmic expression of filamin A was statistically significantly higher in carcinomas compared to atypical adenomas or adenomas [165]. The expression of filamin A also correlated with the serum levels of calcium and PTH, but was not associated with Ki-67 indicating that filamin A plays significant role in calcium turnover but is not associated with the degree of anaplasia [165]. Indeed, Mingione et al., demonstrated that loss of filamin A reduces CaSR expression in protein and mRNA levels; the CaSR-induced ERK phosphorylation also decreases [169].

Filamin A levels are not associated with loss of parafibromin. Pathogenetically, this finding points to different molecular pathways, suggesting diagnostically important conclusion: complex evaluation of both markers might have higher informativity. This hypothesis was proved by Storvall et al., 2021: parafibromin-positive tumours featuring low expression of filamin were likely to be benign [165].

Decreased expression of CaSR in secondary hyperparathyroidism has been demonstrated in an animal model [170] and is associated with hypermethylation of the *CaSR* and *VDR* genes [171]. The down-regulation of CaSR in nodular hyperplasia has been confirmed in human patients, diagnosed with secondary hyperparathyroidism [172]. Tertiary hyperparathyroidism also is associated with lower levers of CaSR [173].

Orphan adhesion G protein-coupled receptor GPR64/ADGRG2 interacts with CaSR. It is overexpressed in parathyroid tumours and attenuates CaSR-mediated signalling [174].

### 3.10. Intratumoural Heterogeneity

Parathyroid tumours are characterised by remarkable biological heterogeneity, involving proliferative activity (Ki-67) and cell cycle regulation (p21, cyclin D1), expression of intermediary filaments (cytokeratin 19, vimentin) and different receptors, e.g., calcium sensing receptor or vitamin D receptor [175]. In addition, technological variations lead to significant intertumoural heterogeneity and differences among data obtained in various studies. The detection of parafibromin is the classic example.

## 4. Tumour Microenvironment

In addition to the gland or tumour parenchyma, represented by the specialised glandular or neoplastic cells themselves, the microenvironment should be accounted for. In neoplasms, the tumour microenvironment is defined as all non-malignant elements present in the tumour that maintain, support, or hinder tumour evolution, for instance, immune and inflammatory cells (tumour-associated lymphocytes, macrophages, neutrophils), endothelial cells along with the cascade of angiogenesis, fibroblasts and myofibroblasts [121]. Many of these cell types and the involved molecular messengers can be detected by immunohistochemistry.

Currently, only few studies have targeted the microenvironment of parathyroid tumours although there are some direct or indirect reports on macrophages [176,177] that were formerly known as a confounding factor in cytology [138,178], and angiogenesis evaluated via morphology or radiological imaging [179,180]. Similarly, scant studies are performed on systemic inflammatory reaction in patients diagnosed with parathyroid tumours [181]. Tumour-infiltrating lymphocytes (TILs) and local immune landscape represent the best explored aspect of parathyroid microenvironment [176,177,182] as these data provide a reliable basis to discuss the applicability of immune therapies [176,183,184].

Programmed death-ligand 1 (PD-L1) in parathyroid tumours has been studied by several research groups [176,182]. On the basis of PD-L1 expression and the presence of TILs, four types of tumour microenvironment have been defined [176]:

- immunotype (IT) I s. adaptive resistance: TILs are present, and PD-L1 is expressed;
- IT II s. immunologic ignorance: both TIL s and PD-L1 are absent;
- IT III s. intrinsic induction: TILs are absent, but PD-L1 is expressed;
- IT IV s. tolerance: TILs are present, but PD-L1 is negative.

The team of Silva-Figueroa et al., 2018 evaluated these immunophenotypes in parathyroid tumours. PD-L1 expression was mostly negative in parathyroid carcinoma, therefore types II and IV predominated (7/18; 38.9% each), followed by type I (3/18; 16.7%). Type III was the least common (1/18; 5.6%) [176]. In a later study, most of parathyroid carcinomas (between 18 and 20 cases out of 26 tumours, depending on the clone of primary antibody) and adenomas (19–25/37) also turned out to be negative [182]. The results were consistent between the studies, but pointed out to technological heterogeneity. The intratumoural density of CD3+, CD8+, CD45+, and CD163+ immune cells in pancreatic carcinoma correlated with disease-free survival [177]. Thus, the evaluation of tumour microenvironment can provide insights into prognosis and immunotherapeutic options (anti-PD-L1 treatment, combination with radiotherapy, vaccines etc.) in accordance with the identified ITs I–IV [176,177]. 

## 5. Conclusions

In conclusion, immunohistochemistry remains an indispensable tool in diagnostic surgical pathology, including parathyroid tumours. In parathyroid pathology, immunohistochemistry has four main applications. First, parafibromin must be detected to identify the new WHO-defined entity, namely, parafibromin-deficient parathyroid neoplasm. Loss of parafibromin indicates greater probability of malignant course and should trigger search for inherited or somatic mutations in *CDC73* gene. Second, a set of markers are down-regulated (e.g., APC, p27 protein, calcium-sensing receptor CaSR) or up-regulated (e.g., proliferation activity by Ki-67 exceeding 5%) in parathyroid carcinoma compared to benign parathyroid disease and thus can be helpful increasing the suspicion of malignancy and prompting the search for the definitive criteria for carcinoma. These criteria are based on morphology: unequivocal invasion or metastasis. Third, upon necessity, immunohistochemistry can be used to find out the histogenesis of a cervical or intrathyroidal mass, or distant metastasis. The differential diagnosis between parathyroid and thyroid tumours, paraganglioma, haematological or metastatic tumours can be clarified via PTH, chromogranin A, TTF-1, calcitonin, LCA, CD56 and vimentin, among others. Finally, immunohistochemistry is a useful tool in pathogenetic studies due to its ability to highlight both the presence and the tissue location of certain proteins. The challenges include technological difficulties (especially, regarding parafibromin stain) and variabilities that might contribute to some of the highlighted controversies. The future developments include both diagnostic and research targets. For practical diagnostics, tumour heterogeneity and technological variations have to be accounted for, finally yielding standardized protocols for staining and evaluation of a unified diagnostic set of immunohistochemical markers. Higher affinity antibodies for parafibromin and menin would be highly desired. Considering pathogenesis of parathyroid tumours, molecular features and tumour microenvironment represent attractive targets.

## Figures and Tables

**Table 1:** Diagnostic criteria for specific types of parathyroid adenomas.

Type	Criterion	Ref.
Oncocytic adenoma	Oncocytes compose >75% of the tumour	Erickson et al., 2022 [12]
Water-clear adenoma	Entirely composed of water-clear cells	
Cystic adenoma	Extensive cystic change affecting >50% of parenchyma	
Lipoadenoma	Stromal fat represents >50% of the tumour	

**Table 2:** Loss of parafibromin expression in parathyroid tumours and tissues.

Pattern	Absolute Numbers of Negative/Investigated Cases; Frequency of Parafibromin Loss (%)					
	Parathyroid Carcinoma	Atypical Parathyroid Tumour	Adenoma	Multiglandular Parathyroid Disease	Normal Gland	Reference
Total nuclear loss	5/5; 100.0%		0/102; 0.0%	1/27 PPH ^1^; 3.7%	0/45; 0.0%	Uljanovs et al., 2021 [38]
Total nuclear loss	2/10; 20.0%	2/46 AA ^2^; 4.3%	2/182; 1.1%			Juhlin et al., 2019 [71]
Partial nuclear loss	5/10; 50.0%	25/46 AA ^2^; 54.3%	8/182; 4.4%			Juhlin et al., 2019 [71]
Nucleolar loss	0/10; 0.0%	3/46AA ^2^; 6.5%	4/182; 2.2%			Juhlin et al., 2019 [71]
Nuclear loss, evaluated via cut-off score	7/21; 33.3%	0/3 AA ^2^; 0.0%	1/73; 1.4%			Hosny Mohammed et al., 2017 [69]
Nuclear loss, defined as <10%	7/14; 50.0%	6/19 AA ^2^; 31.6%	19/194; 9.8%			Kumari et al., 2016 [72]
Total nuclear loss	2/2; 100.0%	0/6 AA ^2^; 0.0%	0/84; 0.0%			Karaarslan et al., 2015 [73]
Total nuclear loss	11/24; 45.8%					Truran et al., 2014 [57]
Total nuclear loss	8/12; 66.7%	2/13 AA ^2^; 15.4%	3/17; 17.6%			Guarnieri et al., 2012 [39]
Total nuclear loss	9/15; 60.0%		1/18; 5.6%	0/8 PH ^3^; 0.0%	0/5; 0.0%	Wang et al., 2012 [67]
Nuclear loss (>99%)	3/8; 37.5%		1/18; 5.6%			Kim et al., 2012 [66]
Total nuclear loss	5/16; 31.3%	0/2 AA ^2^; 0.0%	0/18; 0.0%	0/14 PPH ^1^; 0.0%	0/16 parathyromatosis; 0.0%	Fernandez-Ranvier et al., 2009 [68]
Total nuclear loss	14/27; 51.9%		0/78; 0.0%	0/12 PSTPH ^4^; 0.0%	0/4; 0.0%	Howell et al., 2009 [74]
Total nuclear loss	0/8 ^5^; 0% 1/7 mts; 14.3% mts					Tominaga et al., 2008 [70]
Total nuclear loss	11/11; 100.0%	2/4 AA ^2^; 50.0%	1/22; 4.5%			Cetani et al., 2007 [55]
Total nuclear loss	9/22; 40.9%		0/48; 0%	0/25 PPH ^1^; 0.0%	0/6; 0.0%	Tan et al., 2004 [75]

In the original sources, different terms have been used in accordance with the actual classifications and terminology at the time of publication: ^1^ PPH, primary parathyroid hyperplasia; ^2^ AA, atypical adenoma; ^3^ PH, parathyroid hyperplasia; ^4^ PST PH, primary, secondary or tertiary parathyroid hyperplasia. ^5^ primary and metastatic parathyroid carcinoma in the setting of tertiary hyperparathyroidism, i.e., “on the background of advanced secondary hyperparathyroidism”.

**Table 3:** Proliferation activity by Ki-67 expression in parathyroid tumours and tissues.

Approach to Evaluation	Absolute Numbers of Positive/Investigated Cases; Proportion of Positive Cases (%) or Proliferation Activity by Fraction of Positive Cells (%)					
	Parathyroid Carcinoma	Atypical Parathyroid Tumour	Adenoma	Multiglandular Parathyroid Disease	Normal Gland	Reference
Mean fraction (%) of positive nuclei	5.8%		1.6%	1.0% PPH ^1^	0.4%	Uljanovs et al., 2021 [38]
Hotspot-based nuclear fraction (%)	11.8%		3.5%	2.8% PPH ^1^	1.0%	Uljanovs et al., 2021 [38]
Exceeds cut-off > 5%; NOS	5/10; 50.0%	5/14 AA ^2^; 35,7%				Sungu et al., 2018 [78]
Exceeds cut-off > 5%; NOS	18/21; 85.7%	2/3; 66.7%	0/73; 0.0%			Hosny Mohammed et al., 2017 [69]
Exceeds cut-off 5%; highest *	0/2; 0.0%	1/6 AA ^2^; 16.7%	1/84; 1.2%			Karaarslan et al., 2015 [73]
Exceeds cut-off > 4%; highest	5/23; 21.7%					Truran et al., 2014 [57]
Exceeds cut-off > 5%; NOS	9/15; 60.0%	0/2 AA ^2^; 0.0%	1/18; 5.6%	0/14 PPH ^1^; 0.0%	1/15 parathyromatosis; 6.7%	Fernandez-Ranvier et al., 2009 [68]
Mean fraction of positive nuclei (%)			1.9%	1.8%	3.5%	Kaczmarek et al., 2008 [79]
Exceeds cut-off > 5%; NOS			15/26; 57.7%		0/26; 0.0%	Hadar et al., 2005 [80]
Mean fraction of positive nuclei (%)	2.84% (mean in 17 adenomas and 2 carcinomas)		2.84% (mean in 17 adenomas and 2 carcinomas)	3.38% in 21 PPH ^1^; 3.14% in 30 SPH ^3^	0.19% in 10 normal glands	Thomopoulou et al., 2003 [81]
Mean fraction of positive nuclei (%)	6.1% in 12 carcinomas		3.3% in 11 adenomas	2.6% in 11 hyperplastic glands	0.1 in 9 normal glands	Abbona et al., 1995 [82]

* Authors classified the cases as <1% vs. 1–5 % vs. >5%. Only the latter group is shown here. In the original sources, different terms have been used in accordance with the actual classifications and terminology at the time of publication: ^1^ PPH, primary parathyroid hyperplasia; ^2^ AA, atypical adenoma; ^3^ SPH, secondary parathyroid hyperplasia. NOS, not further specified.

**Table 4:** Technological variables influencing immunohistochemical visualisation.

Step	Variable
Fixation	Time and temperature of cold ischemia before fixation; Choice of the fixative; Time of fixation; underfixation and overfixation
Processing	Protocol of dehydration; Incubation time in xylene and paraffin; Temperature of melted paraffin
Antigen retrieval	Type of antigen retrieval: heat-induced antigen retrieval (HIER) vs. enzymatic treatment vs. none
HIER parameters	Mode: microwave vs. temperature; Temperature, time, pressure (if applicable); pH of the buffer: acidic (e.g., citrate; pH = 6.0), neutral (e.g., TBS; pH = 7.6) vs. basic (e.g., TEG; pH = 9.0)
Primary antibody	Clonality (polyclonal vs. monoclonal), clone, isotype Dilution; Incubation time and temperature
Visualisation system	Choice of the system
Washing of tissue sections	Excessive or insufficient

## Data Availability

Not applicable.
